# Socioeconomic and residence-based inequalities in adolescent fertility in 39 African countries

**DOI:** 10.1186/s12978-024-01806-0

**Published:** 2024-05-31

**Authors:** Bright Opoku Ahinkorah, Richard Gyan Aboagye, Aliu Mohammed, Precious Adade Duodu, Qorinah Estiningtyas Sakilah Adnani, Abdul-Aziz Seidu

**Affiliations:** 1 School of Clinical Medicine, University of New South Wales, Sydney, Australia; 2 REMS Consultancy Services, Takoradi, Western Region Ghana; 3 School of Population Health, University of New South Wales, Sydney, NSW 2052 Australia; 4 Department of Family and Community Health, Fred N. Binka School of Public Health, University of Health and Allied Sciences, Ho, PMB 31, Hohoe, Ghana; 5 Department of Health, Physical Education, and Recreation, University of Cape Coast, Cape Coast, Ghana; 6 Department of Nursing, School of Human and Health Sciences, University of Huddersfield, Queensgate, Huddersfield, England United Kingdom; 7 Department of Public Health, Faculty of Medicine, Universitas Padjadjaran, Bandung, West Java Indonesia; 8 Centre for Gender and Advocacy, Takoradi Technical University, P.O. Box 256, Takoradi, Ghana; 9 College of Public Health, Medical and Veterinary Sciences, James Cook University, Townsville, QLD 4811 Australia

**Keywords:** Adolescent fertility, Inequalities, Residence, Socioeconomic, Africa

## Abstract

**Introduction:**

Despite the advancement in sexual and reproductive healthcare services and several public health measures aimed at controlling fertility rates, countries in sub-Saharan Africa (SSA) still experience higher adolescent fertility rates than other low-and middle-income countries. This study examined the disparities in adolescent fertility in 39 countries in SSA, focusing on socioeconomic and residence-based dimensions.

**Methods:**

This study involved a secondary analysis of data obtained from 39 recent Demographic and Health Surveys conducted in SSA. The measures of difference (D), ratio (R), population attributable fraction (PAF), and population attributable risk (PAR) were estimated using the Health Equity Assessment Tool (HEAT) software version 3.1 developed by the World Health Organization. The measures: D, R, PAF, and PAR were used to examine the inequalities in adolescent fertility across the socioeconomic and residence-based dimensions.

**Results:**

Out of the 39 countries included in the study, Guinea (D=27.70), Niger (D=27.50), Nigeria (D=23.90), and Côte d’Ivoire (D=23.60) exhibited the most significant residence-based inequalities in the rate of adolescent fertility, with the higher rate observed among adolescents in rural areas. Rwanda was the sole country that showed a slight inclination towards rural inequality in terms of the rate of adolescent fertility, with a value of D = -0.80. The burden of adolescent fertility was disproportionately higher among young women with low economic status across all the countries, exacerbating wealth-based inequities. The countries with the largest absolute discrepancies were Nigeria (D=44.70), Madagascar (D=41.10), Guinea (D=41.00), and Cameroon (D=40.20). We found significant disparities in educational attainment contributing to unequal inequalities in adolescent fertility, particularly among young women who lack access to formal education. Countries such as Madagascar (D=59.50), Chad (D=55.30), Cameroon (D=54.60), and Zimbabwe (D=50.30) had the most significant absolute disparities.

**Conclusion:**

This study revealed that young women residing in rural areas, those in households with low economic status and those with limited educational opportunities experience a disproportionately high burden of adolescent fertility across the 39 countries in SSA. The current findings offer valuable information to governmental entities at all levels regarding the need to ensure the provision of equitable, accessible, and dependable sexual and reproductive health services to the populace, particularly for young women. Therefore, the various stakeholders need to enhance the effectiveness of health policies and legislation pertaining to adolescent women living in rural areas, those from economically disadvantaged households, and those with limited or no access to formal education. Such interventions could potentially reduce adolescent fertility rates and mitigate the adverse maternal and child outcomes associated with high adolescent fertility in SSA.

## Plain summary

Adolescent fertility is a major health problem for many developing countries, especially those in sub-Saharan Africa (SSA). Although several sexual and reproductive health initiatives have been introduced in these countries, the number of births among adolescents continues to be high. The present study looked at the socioeconomic and geographical differences in adolescent fertility across 39 countries in SSA using data from the Demographic and Health Surveys embedded into the World Health Organization’s Health Equity Assessment Toolkit (WHO HEAT) software. The study found that in countries like Guinea, Niger, Nigeria, and Côte d’Ivoire, the rates of adolescent fertility varied a lot, with higher rates in rural areas. Generally, poorer young women were more likely to have babies, which made the gap between the rich and the poor even wider. Nigeria, Madagascar, Guinea, and Cameroon had the biggest differences. Education also played a role. In countries like Madagascar, Chad, Cameroon, and Zimbabwe, young women who did not go to school (had no education) were more likely to have children as teenagers.  The study showed that in all 39 countries, young women living in rural areas, those who were poorer and those who did not go to school (had no education) faced a bigger problem with adolescent fertility. The study suggests that if people who make health policies pay more attention to teenage girls in the rural areas, those who are poor and do not have much education, they could make a significant difference in reducing adolescent fertility.

## Introduction

Globally, there has been a substantial decline in adolescent fertility over the past two decades [1, 2]. Adolescent fertility rate (AFR), used as an indicator for adolescent fertility, is the number of births yearly to adolescents aged 15-19 years for every 1,000 women [3, 4]. Adolescent pregnancy is projected to rise worldwide by 2030 due to an expected growth in the total adolescent population [5]. With adolescent pregnancy rates still high in many countries [6], about 21 million girls aged 15–19 years in low-and middle-income countries (LMICs) become pregnant annually [7]. Of this, 50% are unintended and result in about 12 million births [7–9]. Also, 55% of unintended pregnancies among adolescents aged 15–19 years result in unsafe abortions, which are prevalent in LMICs largely due to poor access to information and services on adolescent sexual and reproductive health [7, 8].

There are well-documented adverse outcomes associated with adolescent pregnancies for both adolescent mothers [7, 10] and their babies [7, 11–13]. These adverse maternal and child outcomes increase with decreasing age of the adolescent mother [14, 15]. Furthermore, pregnancy-related complications are major causes of death among adolescent girls globally [15, 16], with approximately 4 million adolescents being at risk of unsafe abortions, which often lead to maternal morbidity and mortality [9]. Also, adolescent pregnancies negatively affect other  adolescent learners, friends, family members, and the community in which the pregnant adolescent belongs [17]. These effects make adolescent pregnancies a major public health concern.

AFR is an adverse health outcome and requires public health interventions to mitigate it [18]. Despite the advancement in sexual and reproductive healthcare services and the implementation of many public health initiatives to control fertility rate, countries in sub-Saharan Africa (SSA) continues to have higher adolescent fertility compared to other LMICs [3, 7, 19]. Globally, SSA has one of the largest population of adolescent girls as well as the highest adolescent birth rate [19–21] and child marriages [22]. In SSA, an estimated 332000 and 6114000 births were recorded among adolescents aged 10–14 years and 15–19 years, respectively, in 2021. Also, an overall adolescent birth rate of 101 per 1000 girls was recorded during the period [19]. However, there are intra-regional variations in AFR. Ahinkorah et al. [23] found that adolescents in certain countries in Africa such as Congo and Chad are more likely to get pregnant than their counterparts in other countries (Rwanda and Burundi). The 2019 World Fertility policy document attributed these inter-country variations in AFR to growth in national wealth, income inequalities, and education [24]. These attributions align with the essence of the Sustainable Development Goal (SDG) target 3.7. This target acknowledges the interrelationship between ensuring universal access to sexual and reproductive healthcare services and poverty eradication due to their linkage with adolescent marriages, pregnancies, and childbirths [25].

Several socioeconomic, cultural, environmental, individual, and health service-related factors are reported as major predictors of adolescent pregnancies and births in SSA [21]. These include sexual coercion, low uptake and use of contraceptives, lack of parental communication and support, early marriage, religion, early sexual debut, rural residence, negative cultural orientation, low self-esteem, and low educational status [21, 26, 27].

Furthermore, inadequate sex education, pressure to marry and bear children, and lack of access to contraception and reproductive health services contribute to adolescent pregnancies [7, 28, 29]. Previous studies in Ghana [30], Ethiopia [31], and across Africa [27] have reported inequalities in AFR, with higher fertility rates recorded among poor adolescent girls, the uneducated, and those residing in rural areas. Thus, maternal educational level, socioeconomic status, and place of residence are critical to AFR, adolescent pregnancies, and their outcomes [32].

Therefore, this study aimed to assess the socioeconomic and residence-based inequalities associated with adolescent fertility in 39 countries in SSA. Analysing data from multiple sources provides comprehensive information on these factors, which could inform policies and interventions aimed at reducing AFR, adolescent pregnancies, and pregnancy-related mortalities and morbidities. Furthermore, this can help to improve the reproductive health and wellbeing of young people in general across the continent. Also, policymakers can utilise our findings  to devise evidence-based interventions to reduce the disproportionately high burden of sexual and reproductive health problems among adolescent girls in SSA [20]. Overall, the findings of this study could contribute towards achieving the maternal and newborn-related SDG 3, specifically, target 3.7, by 2030 [25].

## Methods

### Data source

Data for the study were obtained from the recent Demographic and Health Survey (DHS) of 39 countries in SSA. These were the most recent surveys used in the World Health Organization (WHO)’s Health Equity Assessment Toolkit (HEAT) (Table 1). Over 90 LMICs participated in the DHS globally [33]. The DHS used a cross-sectional design and a two-stage cluster sampling technique to select respondents [34, 35]. Structured questionnaires that have been vetted and approved are used to gather information from respondents. Detailed design and sampling methodology have been highlighted in the literature [33, 34]. This paper was written per the Strengthening the Reporting of Observational Studies in Epidemiology (STROBE) standards [36].

**Table 1:** Residence-based inequality by summary measures across 39 sub-Saharan African countries

**Country**	Year of Survey	D (Estimate)	R (Estimate)	PAF (Estimate)	PAR (Estimate)
**Mali**	2018	13.50	1.50	-26.37	-9.63
**Angola**	2015	13.50	1.39	-9.96	-3.83
**Benin**	2017	9.30	1.70	-28.98	-5.43
**Burkina Faso**	2010	20.30	2.43	-49.64	-14.00
**Burundi**	2016	0.00	1.00	0.00	0.00
**Cameroon**	2018	23.10	2.24	-34.19	-9.66
**Central African Republic**	1994	1.90	1.05	-2.73	-1.03
**Chad**	2014	18.90	1.51	-27.10	-13.72
**Comoros**	2012	6.20	1.48	-25.06	-4.28
**Congo**	2011	16.20	1.64	-14.12	-4.19
**Côte d'Ivoire**	2012	23.60	2.16	-34.37	-10.68
**Democratic Republic of the Congo**	2013	11.30	1.57	-25.38	-6.77
**Eritrea**	2002	18.10	2.19	-40.15	-10.20
**Eswatini**	2006	2.10	1.08	-5.39	-1.51
**Ethiopia**	2019	11.90	1.81	-33.93	-7.55
**Gabon**	2012	18.70	1.72	-6.97	-1.94
**Gambia**	2019	13.60	2.23	-22.78	-3.27
**Ghana**	2014	8.60	1.67	-23.76	-4.02
**Guinea**	2018	27.70	2.17	-39.27	-15.26
**Kenya**	2014	10.70	1.60	-23.72	-5.53
**Lesotho**	2014	3.80	1.33	-17.52	-2.44
**Liberia**	2019	20.20	1.73	-19.24	-6.62
**Madagascar**	2021	19.00	1.95	-41.87	-14.41
**Malawi**	2015	11.20	1.52	-29.71	-9.09
**Mauritania**	2020	19.40	2.45	-40.39	-9.08
**Mozambique**	2011	11.20	1.34	-17.28	-6.94
**Namibia**	2013	7.30	1.60	-18.66	-2.78
**Niger**	2012	27.50	2.05	-45.44	-21.91
**Nigeria**	2018	23.90	2.68	-49.06	-13.68
**Rwanda**	2019	-0.80	0.87	0.00	0.00
**Sao Tome and Principe**	2008	7.40	1.34	-14.35	-3.60
**Senegal**	2018	15.30	3.07	-50.49	-7.55
**Sierra Leone**	2019	16.00	1.68	-24.17	-7.56
**South Africa**	2016	4.70	1.28	-8.45	-1.54
**Togo**	2013	9.10	1.86	-28.66	-4.26
**Uganda**	2016	14.50	1.80	-35.82	-10.16
**United Republic of Tanzania**	2015	11.10	1.72	-30.71	-6.87
**Zambia**	2018	14.90	1.65	-25.13	-7.75
**Zimbabwe**	2015	13.50	1.94	-35.03	-7.71

*D* Difference, *R* Ratio, *PAF *Population attributable, *PAR* Population attributable risk

### Variables

Adolescent fertility was the outcome variable in this study. Young women aged 20-24 who gave birth before their 18^th^ birthday were considered to meet the criteria for adolescent fertility.

We included three inequality stratifiers in our study. These stratifiers: place of residence, educational attainment, and wealth quintile were available in the WHO HEAT software [18, 37]. Both educational attainment and wealth quintile were considered as measures of socioeconomic status. Also, previous studies that utilized the HEAT software included these stratifiers in examining inequalities in several health indicators [30, 38, 39]. The categories of the stratifiers consisted of the following: place of residence (rural, and urban), level of education (no education, primary, secondary, and higher), and wealth quintile (poorest, poorer, middle, richer, and richest).

### Analyses

The online version of WHO’s HEAT software [37] was used to perform the analysis. HEAT is a statistical online software designed to examine health inequalities within and between countries on several health indicators and social issues encompassing child, maternal, and reproductive health [37]. The detailed description of HEAT can be found in the literature [37]. We considered four inequality measures in this study. These measures consisted of difference (D), ratio (R), population attributable fraction (PAF), and population attributable risk (PAR). Of these measures, R and PAF are called relative summary measures, whilst the remaining two are absolute summary measures. Both D and R are simple measures and PAR and PAF are complex measures. Detailed significance, calculation, and interpretation of these four measures have been highlighted in the literature [37, 40]. Briefly, the value of zero (0) for D signifies no inequality whilst higher values show higher inequality in adolescent fertility. The higher values of R correspond to higher concentrations of adolescent fertility. R takes on the value 1 if there is no inequality. Only positive values are accepted. The degree of inequality increases when R's value deviates from 1. PAF and PAR take positive values for favourable indicators and negative values for adverse indicators. The larger the absolute value of PAF and PAR, the larger the level of inequality. PAF and PAR are zero if no further improvement can be achieved, i.e. if all subgroups have reached the same level of the indicator as the reference subgroup.

### Ethical considerations

Ethical clearance was not sought for this study because we analysed a secondary data, which is freely available in the public domain. The detailed ethical issues concerning the DHS can be accessed via https://dhsprogram.com/Methodology/Protecting-the-Privacy-of-DHS-Survey-Respondents.cfm.

## Results

### Residence-based inequalities in adolescent fertility in sub-Saharan Africa

**Fig. 1:**
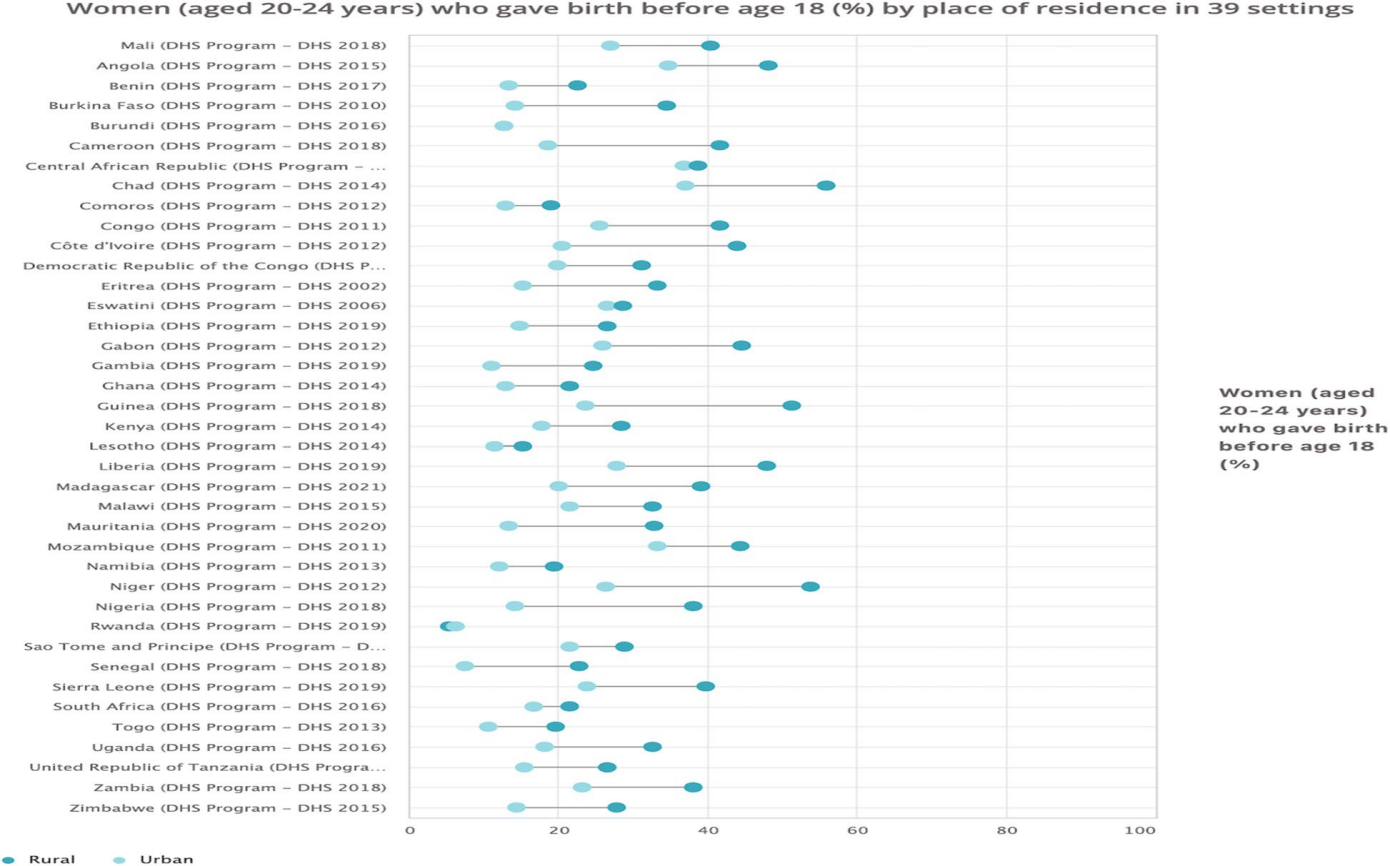
Women (aged 20-24 years) who gave birth before age 18 years (%) by place of residence in 39 countries

Figure 1 shows the results of the residence-based inequalities in adolescent fertility in SSA. For all the 39 countries surveyed, adolescent fertility was higher in rural areas compared to urban areas. This was different in Rwanda, where adolescent fertility was higher in urban areas compared to rural areas. Chad was the country with the highest adolescent fertility in both rural and urban areas while Rwanda had the least prevalence of adolescent fertility in both rural and urban areas.

Tables 1 shows the extent of residence-based inequalities in adolescent fertility across the countries in SSA. Both simple (D, R) and complex (PAF, PAR) summary measures revealed substantial disparities in adolescent fertility in all the dimensions of inequality, with higher disparities observed among young women who lived in rural areas, those with low economic status, and those who had no formal education. For example, in the residence-based inequality, using simple measures of inequality, the findings showed high inequality (D, R) in adolescent fertility with higher disparities recorded among young women in rural areas. Among the 39 countries surveyed, Guinea (D=27.70), Niger (D=27.50), Nigeria (D=23.90), and Côte d’Ivoire (D=23.60) recorded the highest levels of disparities. Only Rwanda showed marginal pro-rural inequality in adolescent fertility (D = -0.80). These patterns were also observed using the complex measures of inequality (PAF, PAR). For instance, the PAR values revealed wide residence-based inequalities in adolescent fertility in most of the countries surveyed, with lower disparities observed among adolescents in urban areas, notably, in countries like Niger (PAR = -21.91), Guinea (PAR = -15.26), Madagascar (PAR = -14.41), Burkina Faso (PAR = -14.00), Chad (PAR = -13.72), and Nigeria (PAR = -13.68). These PAR values suggest that in the absence of residence-based disparities, the setting average of adolescent fertility would have decreased by 21.91%, 15.26%, 14.41%, 14.00%, 13.72%, and 13.68% in Niger, Guinea, Madagascar, Burkina Faso, Chad, and Nigeria, respectively.

### Wealth-based inequalities in adolescent fertility in sub-Saharan Africa

**Fig. 2:**
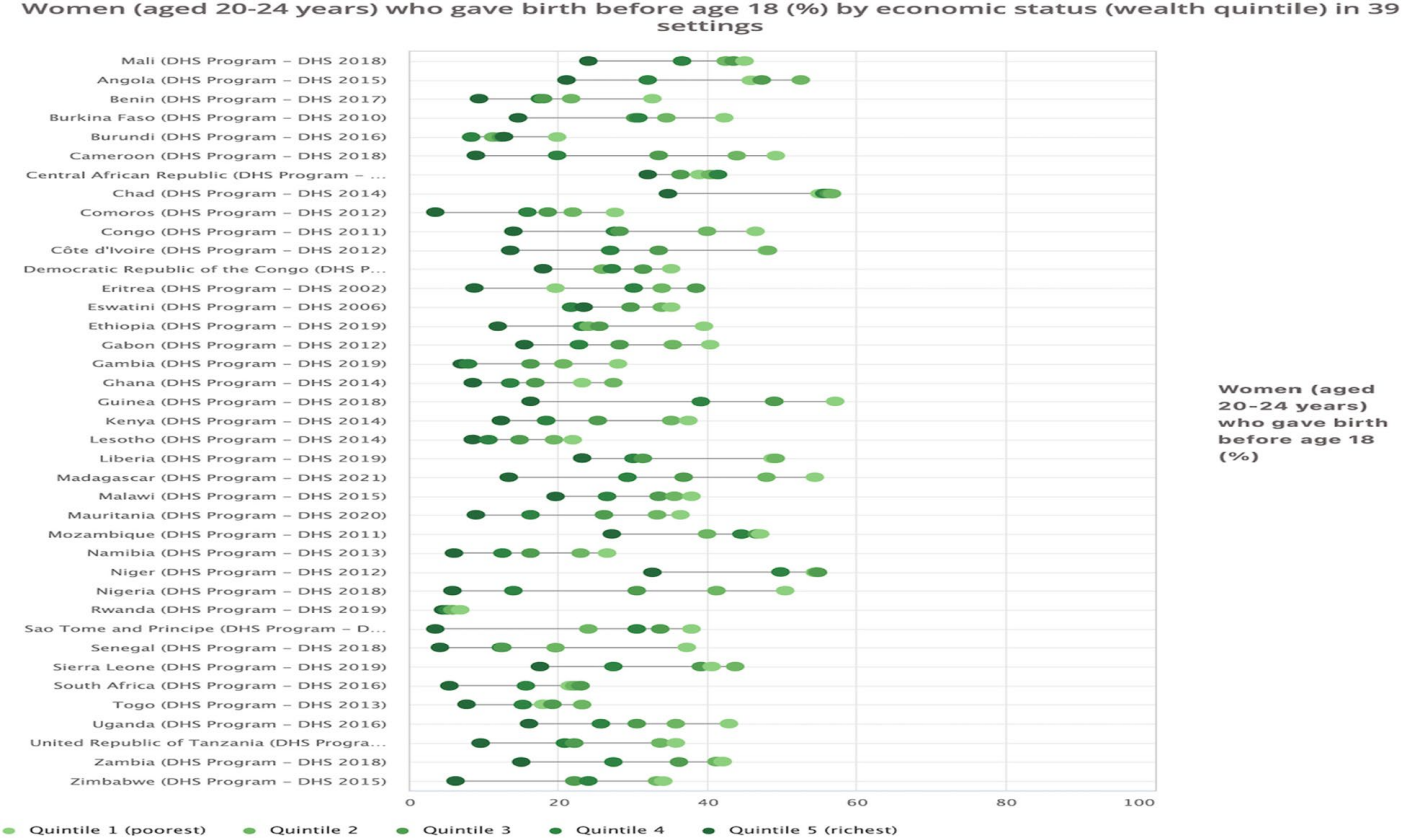
Women (aged 20-24 years) who gave birth before age 18 years (%) by economic status (wealth quintile) in 39 countries

Figure 2 shows the results of the wealth-based inequalities in adolescent fertility in SSA. For all the 39 countries surveyed, adolescent fertility was higher among adolescents in households with the poorest wealth quintile and lowest in households with the richest wealth quintile. Guinea had the highest prevalence of adolescent fertility among young women in the poorest wealth quintile, while Comoros had the lowest fertility among young women in the richest wealth quintile.

As shown in Table 2, we found simple (D, R) and complex (PAF, PAR) wealth-based inequalities in the burden of adolescent fertility with higher disparities observed among young women with low economic status in all the countries surveyed. However, the widest disparities using the simple measure of D were observed in Nigeria (D=44.70), Madagascar (D=41.10), Guinea (D=41.00), and Cameroon (D=40.20). Meanwhile, the PAR measure revealed wide wealth-based inequality in adolescent fertility with lower disparities observed among young women with higher economic status. Countries with the greatest disparities include Guinea (PAR = -22.65), Nigeria (PAR = -22.07), Sao Tome and Principe (PAR = -21.67), Madagascar (PAR = -21.00), and Cameroon (PAR = -19.35). The PAR measures suggest that in the absence of the disparities in wealth among the population, the setting average of adolescent fertility could decline by 22.65%, 22.07%, 21.67%, 21.00%, and 19.35% in Guinea, Nigeria, Sao Tome and Principe, Madagascar, and Cameroon, respectively.

**Table 2:** Wealth-based inequality by summary measures across 39 sub-Saharan African countries

**Setting**	Year	D (Estimate)	R (Estimate)	PAF (Estimate)	PAR (Estimate)
**Mali**	2018	21.00	1.88	-34.58	-12.63
**Angola**	2015	24.80	2.18	-45.29	-17.38
**Benin**	2017	23.40	3.52	-50.36	-9.43
**Burkina Faso**	2010	27.60	2.89	-48.21	-13.59
**Burundi**	2016	7.30	1.58	-0.85	-0.11
**Cameroon**	2018	40.20	5.52	-68.50	-19.35
**Central African Republic**	1994	7.00	1.22	-15.43	-5.82
**Chad**	2014	20.40	1.59	-31.39	-15.87
**Comoros**	2012	24.10	8.09	-80.04	-13.63
**Congo**	2011	32.50	3.34	-53.18	-15.79
**Côte d'Ivoire**	2012	34.30	3.52	-56.27	-17.50
**Democratic Republic of the Congo**	2013	17.20	1.96	-32.88	-8.77
**Eritrea**	2002	10.70	2.22	-65.45	-16.67
**Eswatini**	2006	11.70	1.50	-16.41	-4.59
**Ethiopia**	2019	27.70	3.35	-46.96	-10.45
**Gabon**	2012	25.00	2.62	-44.56	-12.38
**Gambia**	2019	20.80	3.93	-50.52	-7.25
**Ghana**	2014	14.70	2.75	-50.22	-8.48
**Guinea**	2018	41.00	3.53	-58.30	-22.65
**Kenya**	2014	25.20	3.07	-47.62	-11.09
**Lesotho**	2014	13.50	2.61	-39.68	-5.53
**Liberia**	2019	25.50	2.10	-32.64	-11.24
**Madagascar**	2021	41.10	4.07	-61.05	-21.00
**Malawi**	2015	18.10	1.92	-35.67	-10.92
**Mauritania**	2020	27.50	4.09	-60.45	-13.60
**Mozambique**	2011	19.90	1.73	-32.28	-12.97
**Namibia**	2013	20.70	4.51	-60.34	-8.98
**Niger**	2012	21.80	1.67	-32.20	-15.53
**Nigeria**	2018	44.70	8.71	-79.19	-22.07
**Rwanda**	2019	2.40	1.53	-17.95	-0.98
**Sao Tome and Principe**	2008	34.50	11.15	-86.44	-21.67
**Senegal**	2018	33.20	9.30	-73.17	-10.91
**Sierra Leone**	2019	22.90	2.30	-43.68	-13.65
**South Africa**	2016	16.20	4.06	-70.97	-12.96
**Togo**	2013	10.20	2.32	-48.24	-7.18
**Uganda**	2016	26.90	2.68	-43.58	-12.36
**United Republic of Tanzania**	2015	26.10	3.72	-57.11	-12.78
**Zambia**	2018	27.20	2.83	-51.76	-15.99
**Zimbabwe**	2015	27.80	5.48	-71.83	-15.81

*D* Difference, *R* Ratio, *PAF* Population attributable fraction, *PAR* Population attributable risk

### Education-based inequalities in adolescent fertility in sub-Saharan Africa

**Fig. 3:**
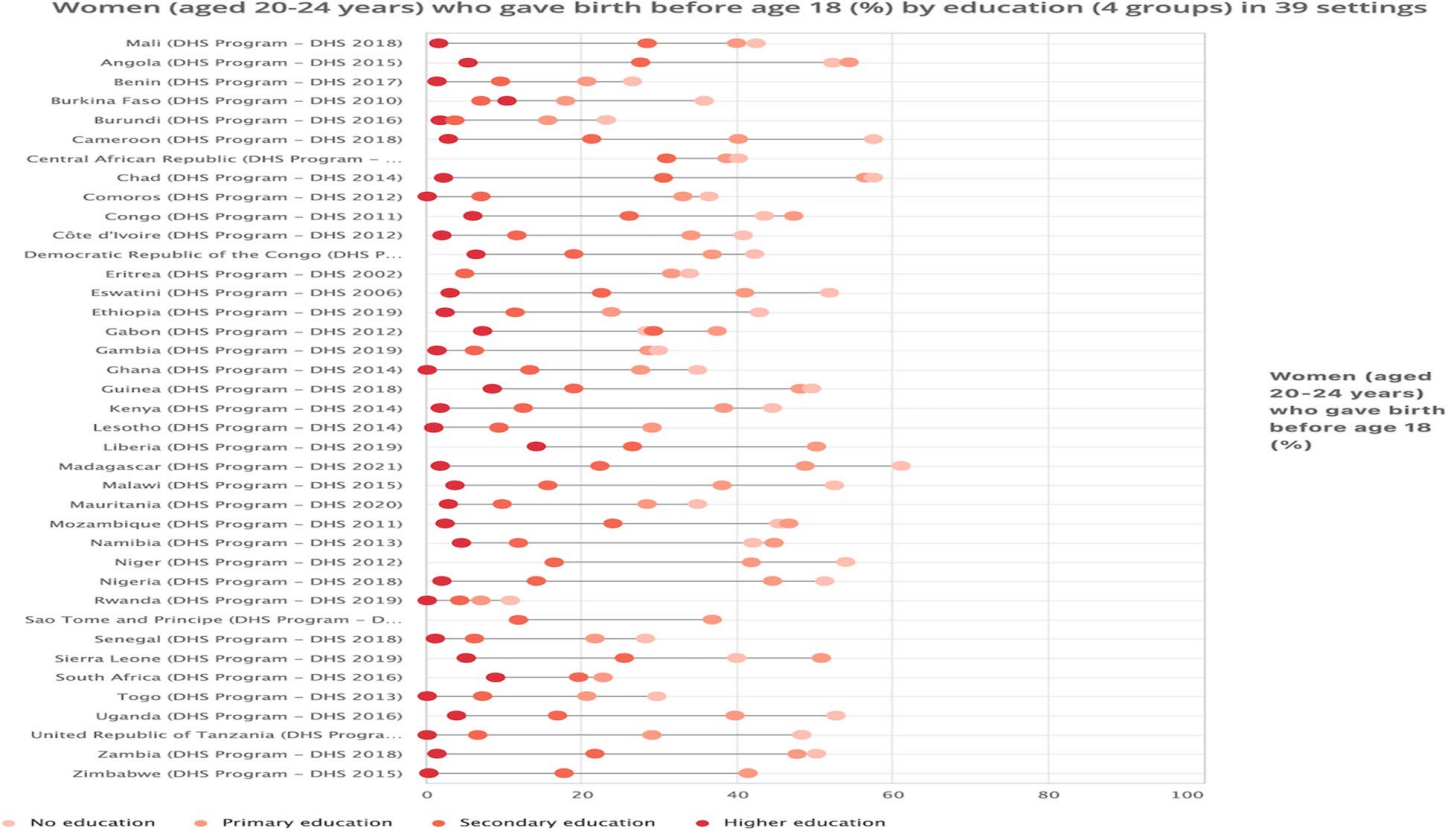
Women (aged 20-24 years) who gave birth before age 18 years (%) by education in 39 countries

As shown in Fig. 3, adolescent fertility was highest among young women with no formal education in all the 39 African countries surveyed. Madagascar recorded the highest prevalence of adolescent fertility among young women with no formal education, while Rwanda had the lowest adolescent fertility among young women with higher education.

As shown in Table 3, we observed wide education-based inequalities in adolescent fertility, with higher disparities observed among women who have no formal education. The widest disparities using a simple measure of D were seen in countries like Madagascar (D=59.50), Chad (D=55.30), Cameroon (D=54.60), and Zimbabwe (D=50.30). Also, the PAR measure showed high education-based inequalities in adolescent fertility with lower disparities seen among young women with higher education, especially in countries like Chad (PAR = -48.44), Mozambique (PAR = -37.79), Mali (PAR = -35.04), Angola (PAR = -32.98), and Madagascar (PAR = -32.67). These PAR values suggest that without education-based disparities, the the setting average of adolescent fertility could decline by 48.44%, 37.79%, 35.04%, 32.98%, and 32.67% in Chad, Mozambique, Mali, Angola, and Madagascar, respectively.

**Table 3:** Education-based inequality by summary measures across 34 African countries

**Setting**	Year	D (Estimate)	R (Estimate)	PAF (Estimate)	PAR (Estimate)
**Mali**	2018	41.00	28.33	-95.90	-35.04
**Angola**	2015	47.00	9.70	-85.93	-32.98
**Benin**	2017	25.20	19.00	-92.53	-17.34
**Burkina Faso**	2010	25.50	3.48	-63.47	-17.90
**Burundi**	2016	21.40	13.59	-86.60	-10.98
**Cameroon**	2018	54.60	19.83	-89.72	-25.30
**Chad**	2014	55.30	26.14	-95.66	-48.44
**Comoros**	2012	36.40	N/A	-100.00	-17.01
**Congo**	2011	37.60	7.37	-80.15	-23.82
**Côte d'Ivoire**	2012	38.80	20.40	-93.57	-29.09
**Democratic Republic of the Congo**	2013	36.00	6.71	-76.38	-20.37
**Eswatini**	2006	48.80	17.27	-89.28	-24.98
**Ethiopia**	2019	40.50	17.87	-89.23	-19.87
**Gabon**	2012	21.20	3.90	-73.75	-20.50
**Gambia**	2019	28.50	22.92	-90.94	-13.06
**Ghana**	2014	35.00	N/A	-100.00	-16.90
**Guinea**	2018	41.20	5.90	-78.38	-30.45
**Kenya**	2014	42.80	24.78	-92.28	-21.53
**Liberia**	2019	36.10	3.56	-59.07	-20.35
**Madagascar**	2021	59.50	36.00	-95.05	-32.67
**Malawi**	2015	49.00	14.61	-88.24	-27.02
**Mauritania**	2020	32.20	12.50	-87.53	-19.65
**Mozambique**	2011	42.90	18.87	-94.03	-37.79
**Namibia**	2013	37.40	9.13	-69.02	-10.25
**Nigeria**	2018	49.20	25.60	-92.82	-25.86
**Rwanda**	2019	10.80	N/A	-100.00	-5.48
**Senegal**	2018	27.00	23.50	-91.96	-13.72
**Sierra Leone**	2019	34.70	7.67	-83.35	-26.04
**South Africa**	2016	29.70	4.54	-58.63	-11.91
**Togo**	2013	29.60	N/A	-100.00	-14.82
**Uganda**	2016	48.90	13.87	-86.59	-24.55
**United Republic of Tanzania**	2015	48.30	N/A	-100.00	-22.40
**Zambia**	2018	48.90	35.93	-95.46	-29.46
**Zimbabwe**	2015	50.30	5.70	-46.17	-9.18

*N/A* Not Applicable, *D* Difference, *R* ratio, *PAF* Population attributable fraction, *PAR* Population attributable risk

Note: Data was not available to calculate the summary measures for five countries (Central African Republic, Eritrea, Lesotho, Niger, and Sao Tome and Principe)

## Discussion

The present study revealed substantial socioeconomic (wealth and education) and residence-based disparities in adolescent fertility, with higher inequalities observed among young women who resided in rural areas, those with low economic status, and those with no formal education. These disparities were observed in all the countries surveyed albeit at varying degrees, suggesting a persistent need for country-specific interventions to address the problem of high AFR in SSA.

Several previous studies reported a significant association between AFR and the dimensions of inequality including rural-urban residency, wealth, and education [30, 42–44]. In this study, we observed varied residence-based disparities in adolescent fertility, which skewed towards young women living in rural areas in all the countries surveyed except in Rwanda, where young women in urban areas had a marginally higher burden of adolescent fertility. This finding supports previous studies that have reported that adolescents who reside in rural areas have a higher fertility rate relative to those in urban areas [27, 44, 45]. In Ethiopia for instance, Alemayehu et al. [46] found that adolescents living in rural areas were four times more likely to have children than those in urban areas. The high fertility rate among adolescents in rural areas has been attributed to poverty and lack of educational opportunities [47], as well as limited access to sexual and reproductive health information and services [43]. Meanwhile, aside from having better access to contraceptives such as condoms, adolescents in urban areas are more exposed to social norms that discourage early marriages and childbirth, which contribute to their reduced fertility rate [42].

Remarkably, our observation in Rwanda supports earlier studies which reported that Rwanda has the lowest AFR in SSA [23, 43], with young women in urban areas having marginally higher rates of adolescent fertility [48]. The declining rate of adolescent fertility in Rwanda, particularly in rural areas, has been attributed to the persistent implementation of public interventions such as increased sex education, sexual and reproductive health promotion, and monitoring of girl-child education [48]. Other interventions include an enhanced legal framework to punish men who impregnate young girls [48], increased access to modern contraceptives, and improved family planning services [49]. Meanwhile, we observed that most of the countries with the greatest simple (Guinea, Niger, Nigeria, and Côte d’Ivoire), and complex (Niger, Guinea, Madagascar, Burkina Faso, Chad, and Nigeria) residence-based disparities in adolescent fertility were from West Africa. The high fertility rate among young women in West Africa was reported in previous studies [43, 45]. Thus, efforts to address the problem of high AFR in SSA must pay particular attention to countries in West Africa, especially among their rural population.

Similar to the findings from earlier studies [44, 50, 51], we observed varied wealth-based disparities in the rate of adolescent fertility, with lower rates observed among young women with the highest wealth quintile across all the countries surveyed. Both complex (PAR) and simple (D) inequality dimensions revealed that countries like Guinea, Nigeria, Madagascar, Cameroon, and Sao Tome and Principe ranked high in wealth-based disparities in adolescent fertility in SSA. Meanwhile, available evidence suggests that countries with increased wealth-based disparities have the highest AFR globally [3]. The increased rate of adolescent fertility among the poor has been attributed to their limited ability to access reproductive healthcare services including family planning [52], dropping out of school, increased exposure to early sexual debut [53], and increased societal pressure to get married and start childbearing [51, 54]. Thus, our findings affirm the need for persistent efforts to reduce poverty and close the income inequality gaps across the countries in SSA, particularly among those with high AFR.

Consistent with several previous studies [30, 44, 46], our findings revealed that wide disparities exist in adolescent fertility based on young women’s level of education, with lower burden observed among those with higher educational attainment. For instance, the complex (PAR) inequality measures suggest that in the absence of education-based disparities in the population, AFR would have reduced by 48.44%, 37.79%, 35.04%, 32.98%, and 32.67% in Chad, Mozambique, Mali, Angola, and Madagascar, respectively. Increased access to education reduces the risk of early sexual debut [53], early marriage and childbirth [54], and increased use of reproductive health services such as modern contraceptives [55, 56], which reduce AFR. Meanwhile, access to secondary or higher education remains poor in many countries in SSA although that of primary education has largely improved [57, 58]. For instance, Ilie and Ros [57] reported that the higher education net attendance rate in countries like Mozambique, Madagascar, and Mali is below 5%. Perhaps, increasing access to higher education and bridging the educational inequality gap could significantly reduce AFR in SSA, particularly in countries like Chad, Mozambique, Mali, Angola, and Madagascar.

### Practical implications

Findings from this study suggest that the rate of adolescent fertility is disproportionately higher among women in rural areas, those with low economic status, and those with no or less formal education across the countries in SSA. Since the prevalence of adolescent fertility tends to be lower in countries with the lowest disparity in the dimensions of inequality [3], strategies aimed at reducing AFR in SSA could be targeted at bridging the inequality gaps in residence, wealth, and education across the countries. For example, increasing adolescents’ access to sexual and reproductive health information and services in rural areas, providing economic opportunities and financial support to less privileged adolescents, and implementing policies to improve female access to education and monitoring their educational progression could contribute towards reducing the burden of adolescent fertility among the disadvantaged population. Perhaps, such interventions could contribute toward the realization of the 2030 SDG agenda [59], and the United Nation’s global strategy for women’s, children’s, and adolescent health [60]. Also, because the present study provides multi-country data on inequality dimensions and the burden of adolescent fertility in SSA, it allows for a comparison of the disparities across the countries studied. Thus, our findings highlight the urgency for interventions to address the high rate of adolescent fertility, especially among countries like Guinea, Niger, Nigeria, Chad, Mali, Côte d’Ivoire, Madagascar, Burkina Faso, Madagascar, Cameroon, and Sao Tome and Principe. Besides, the multi-country analysis also provides data for progress monitoring in future studies.

### Strengths and limitations

In this study, we employed nationally representative datasets to provide insight into the socioeconomic and residence-based inequalities in adolescent fertility in SSA. As a result, our findings provide a foundation for tracking differences in the burden of AFR among sub-Saharan African countries using the WHO's HEAT software. Despite these strengths, the current study has drawbacks. First, the DHS datasets included in this study were done at different times in different countries. This may induce bias when comparing the extent of differences among countries in the dimensions of inequality and the rate of adolescent fertility. Aside from the varying survey dates, our study was based on a single survey year in each country. Hence, we were unable to conduct a trend analysis to ascertain the pattern of inequality characteristics in adolescent fertility across the countries over time. Finally, because both the inequality dimensions and the adolescent fertility were self-reported, they could be susceptible to recall and social desirability biases.

## Conclusion

This study highlights the inequalities in adolescent fertility in SSA. Our findings revealed that young women who live in rural areas, those with low economic status, and those who had no formal education were disproportionately disadvantaged in the rate of adolescent fertility across the countries. Thus, interventions aimed at reducing AFR in SSA should be targeted at young women who reside in rural areas, those from poor households, and those with lower or no formal education. This could contribute towards reducing AFR and its associated poor maternal and child outcomes in SSA as well as the realization of the SDG target 3.7.

## Data Availability

The dataset used can be accessed at https://dhsprogram.com/data/available-datasets.cfm.

## Abbreviations

AFR: Adolescent Fertility Rate
D: Difference
DHS: Demographic and Health Survey
HEAT: Health Equity Assessment Toolkit
LMICs: Low-and Middle-Income Countries
PAF: Population Attributable Fraction
PAR: Population Attributable Risk
R: Ratio
SDG: Sustainable Development Goal
SSA: Sub-Saharan Africa
STROBE: Strengthening the Reporting of Observational Studies in Epidemiology
WHO: World Health Organization
